# Etiology of Multiple Non-EV71 and Non-CVA16 Enteroviruses Associated with Hand, Foot and Mouth Disease in Jinan, China, 2009—June 2013

**DOI:** 10.1371/journal.pone.0142733

**Published:** 2015-11-12

**Authors:** Hengyun Guan, Ji Wang, Chunrong Wang, Mengjie Yang, Lanzheng Liu, Guoliang Yang, Xuejun Ma

**Affiliations:** 1 Viral Disease Inspection Laboratory, Jinan Municipal Center for Disease Control and Prevention, Jinan, China; 2 Key Laboratory of Medical Virology, Ministry of Health, National Institute for Viral Disease Control and Prevention, Chinese Center for Disease Control and Prevention, Beijing, China; Singapore Immunology Network, Agency for Science, Technology and Research (A*STAR), SINGAPORE

## Abstract

Hand, foot, and mouth disease (HFMD) is an infectious disease caused by human enterovirus 71 (EV71), coxsackievirus A16 (CVA16) and other enteroviruses. It is of interest that other enteroviruses associated with HFMD in Jinan have been rarely reported. The aim of the present study is to detect and characterize the circulating serotypes of non-EV71 and non-CVA16 enteroviruses associated with HFMD in Jinan city, Shandong province, China. A total of 400 specimens were collected from clinically diagnosed HFMD cases in Jinan from January 2009 to June 2013. All specimens were infected with non-EV71 and non-CVA16 enteroviruses previously confirmed by RT-PCR or real-time PCR according to the protocols at that time. The GeXP-based multiplex RT-PCR assay (GeXP assay) was performed to investigate the pathogen spectrum of 15 enteroviruses (coxsackieviruses A4, A5, A6, A9, A10, A16; coxsackieviruses B1, B3, B5; Echoviruses 6, 7, 11, 13, 19 and EV71) infections associated with HMFD. For GeXP assay negative samples, reverse transcription nested PCR (nested RT-PCR) based on the 5’ -untranslated region (5’- UTR) sequence and phylogenetic analysis were conducted to further explore the etiology of multiple enteroviruses. The results showed that a total of twenty serotypes of enteroviruses (including EV71 and CVA16) were identified by GeXP assay and nested RT-PCR. The most circulating twelve serotypes of enteroviruses with HFMD in Jinan from 2009 to June 2013 were EV71, CVA16, CVA10, CVA6, CVA12, CVA2, Echo3, CVA4, CVA9, CVB1, CVB3 and Echo6. CVA10 and CVA6 were the most prevalent pathogens other than EV71 and CVA16 in Jinan and their most prevalent seasons were spring and summer, and a slight increase was observed in autumn and early winter. It should be noted that mixed-infections were identified by GeXP assay and the phylogenetic tree clearly discriminated the multiple pathogens associated with HFMD. Our results thus demonstrate that there was a clear lack of a reliable testing method for EV71 and CVA16 and multiple non-EV71 and non-CVA16 enteroviruses associated with HFMD were present in Jinan. The GeXP assay combined with nested RT-PCR based on 5’-UTR region could meet the need for the national surveillance of multiple enteroviruses or the investigation of epidemic outbreaks triggered by enteroviruses in the future.

## Introduction

Hand, foot and mouth disease (HFMD) is a prevalent, typically self-limited viral syndrome in children and adults. It has emerged as a major public health concern in China since the first case of HFMD was reported in Shanghai in 1982 [1]. The mild presentations are marked by fever, oral ulcers and papules on the palms of the hands, the soles of the feet and buttocks. In some cases, severe complications can occur, including central neurological and cardiopulmonary manifestations such as encephalomyelitis, aseptic meningitis, acute flaccid paralysis, pulmonary edema, end-organ dysfunction and even death [2].

HFMD has been found to be associated with various enteroviruses including human enterovirus 71 (EV71), coxsackievirus A(1–10, 12, 16, 22) and coxsackievirus B(1–5) and echoviruses [3–7]. EV71 and CVA16 were still the leading cause of HFMD in Jinan, the capital city of Shandong province. In addition to high rates of EV71 and CVA16 infections, other enteroviruses have also emerged as major pathogens based on national surveillance [8–9] such as coxsackievirusA12 emerged in Qingdao during 2008–2012 [10], outbreaks of coxsackievirus A10 and A3 in Shijiazhuang during 2010–2012 [11] and circulation of coxsackievirus A6 in Guangzhou [6]. In addition, the mixed-infections of EV71 or CVA16 with other enteroviruses were reported and resulted in the worse condition and longer courses for patients compared to a single enterovirus infection (EV71 or CVA16) [12–14]. However, the role of other enteroviruses in mixed-infections is not very clear due to the lack of accurate diagnosis. Virus isolation and immunofluorescence assay are traditional and time-consuming methods for detection and serotyping of enteroviruses. Classic molecular tests like RT-PCR or real-time PCR are difficult to identify multiple serotypes in one tube. GeXP multiplex assay (GeXP assay) allows for a high throughput, robust, and differential detection of up to 35 PCR products based on size by capillary gel electrophoresis and has been successfully used in our laboratory to develop diagnostic microarrays for sixteen human respiratory viruses [15], eleven human papillomaviruses [16] and nine serotypes of enteroviruses associated with HFMD [17]. In this study, GeXP assay was further applied to simultaneously detect fifteen common serotypes of enteroviruses, including coxsackieviruses (A4, A5, A6, A9, A10, A16); coxsackieviruses (B1, B3, B5); echoviruses (6, 7, 11, 13, 19) and EV71. For GeXP assay negative samples, nested RT-PCR [18] and phylogenetic analysis based on 5’-UTR region were then further performed to fully explore the pathogen spectrum and epidemiology of enteroviruses associated with HFMD in Jinan over the period from January 2009 to June 2013.

## Materials and Methods

### Sample collection

There are ten districts in Jinan and every district has set up sentinel sites responsible for national surveillance program for HFMD. A total of 400 samples were obtained from patients who were clinically diagnosed with HFMD at sentinel hospitals, including 391 stool samples and 9 throat swabs from 2009 to June 2013. The diagnostic criteria of HFMD were defined by Ministry of Health. These samples were tested negative for EV71, CVA16, and positive for pan-enteroviruse using classic methods [8–9].

### Pretreatment of samples and total RNA isolation

Stool samples (2g) were dissolved in 1ml of phosphate buffer saline (with Mg^2+^ and Ca^2+^) and vortex for 2×20 sec at 4000rpm, then centrifuged at 8000 rpm for 10 min. The supernatant was collected and used for RNA extraction. Throat swabs were not pre-treated prior to extraction. Total viral nucleic acid was extracted from 200μl of stool supernatant or throat swab using the MagNA Pure LC Total Nucleic Acid Isolation Kit (Roche, Germany) according to the manufacturer’s instructions. 50μl eluted solution was stored at −80°C.

### Multiplex RT-PCR and Fragment analysis by GenomeLab GeXP Genetic Analysis System

To expand the pathogen spectrum reported previously in our laboratory [17], two panels of primer mixtures were constructed for multiplex RT-PCR (mRT-PCR) assay. Panel A consisted of 9 pairs of chimeric primers (including one pair of pan-enterovirus primers and 8 pairs of human enterovirus serotype-specific primers for EV71, CVA16, CVA5, CVA9, CVA10, CVB1, CVB3 and CVB5, respectively) and one pair of universal primers (Tag-F/Tag-R). Panel B consisted of 8 serotype-specific primers for EV71, CVA16, CVA4, CVA6, Echo6, Echo 7, Echo 11 and Echo 19 in addition to pan-enterovirus primers and universal primers. The mRT-PCR assay was conducted using One Step RT-PCR Kit (Qiagen, Germany) in a 25μl volume containing 1 μl of extracted viral RNA from each samples, 1.25 μl of gene-specific chimeric primer mixtures (final concentrations 50 nM) and 1.25 μl of universal tag primers (final concentrations 500 nM). The mRT-PCR assay with three steps of amplification was carried out in a thermal cycler as described previously [17]. A positive and a negative control were both set up as the quality control for enterovirus detection.

mRT-PCR products were separated and detected by a GenomeLab GeXP Genetic Analysis System (Beckman Coulter, USA) in accordance with the manufacturer’s instructions. Briefly, 2.0 μl of each PCR product was blended with the sample loading solution (SLS) mixtures and analyzed on GeXP genetic analysis system. The specific DNA fragment peaks were observed on the GeXP analyzer and matched to the appropriate genes.

### Reverse transcription-nested PCR and sequencing on 5’-UTR region

To further identify the etiology of enteroviruses causing HFMD, reverse transcription-nested PCR (nested RT-PCR) with pan-enterovirus specific primers targeting the 5’-UTR region [18] was used to further screen GeXP assay negative samples. Briefly, the SuperScript^TM^ III One-Step RT-PCR System (Invitrogen, USA) was used for the first RT-PCR containing 0.5 μl of 10 mM outer primers. The RT-PCR was carried out on a thermal cycler (Thermo Electron, USA) under the conditions reported in previous study [18]. The One Step Kit (TaKaRa, Japan) was used for the second PCR containing 0.5 μl of 10 mM inner primers and 1 μl product of first RT-PCR. There was no reverse transcription step compared to the first RT-PCR procedures for the reaction conditions. Negative controls (sterile water) were constructed in each reaction.

### Sequence Analysis

The nested RT-PCR yielded about a 389bp amplicon. The final PCR products were subjected to sequencing in both directions using the ABI 3730 XL DNA Analyzer (Applied Biosystem Inc., Foster City, CA). Nucleotide sequences of 5’-UTR were checked by the BLAST search in the NCBI database to identify the enterovirus serotype with the highest identity. Obtained sequences were assembled using SeqMan software (version7.1.0). Phylogenetic tree was constructed by neighbor-joining method with 1000 bootstrap replications using MEGA6.0. Reference sequences representing EV71, CVA (2, 4, 6, 10, 12, 16), CVB4, Echo (3, 9, 25) and HEV-C were downloaded from NCBI database and selected for phylogenetic analysis.

### Ethics statement

This study was approved by the Ethics Committee at Jinan Municipal Center for Disease Control and Prevention, China. Written informed consent was obtained from the parents of every child participant and non minor/child participants enrolled in this study.

## Results

### Cases and Etiology

From January 2009 to July 2013, a total of 400 specimens were tested in this study. The age of these cases ranged from 8 months to 21 years with 72.25% (289 children) being less than 3 years old, and 22.75% (91 children) being 4 to 5 years old. The male-to-female ratio was 1.52:1.

**Fig 1:**
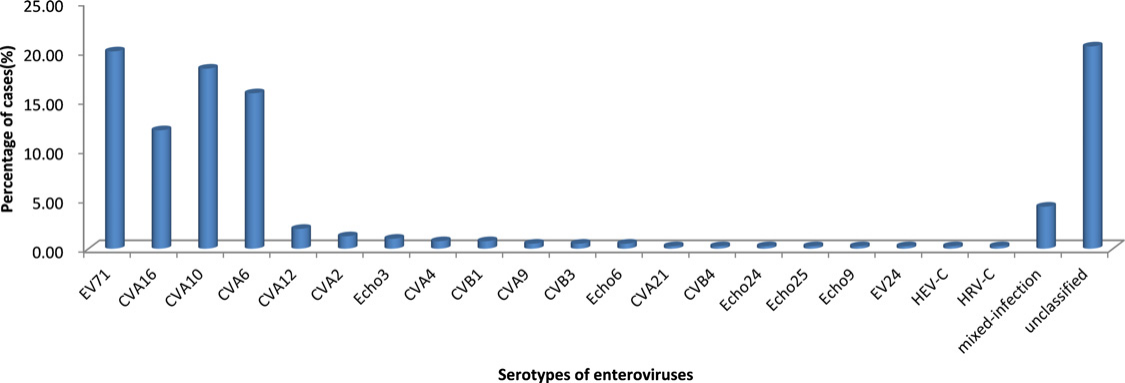
The enterovirus serotypes identified in hand-foot-mouth disease during 2009 to June 2013, in Jinan, China.

A total of 293 (73.25%) cases were identified by GeXP assay and the true positives (GeXP-positive) for EV71, CVA16, CVA6, CVA10 and CVA4 were confirmed by nested RT-PCR with pan-enterovirus specific primers targeting the 5’-UTR region [18]. Additionally the serotypes of 25(7.86%) GeXP-negative specimens were verified by 5’-UTR amplification and sequencing. However, 82 (20.50%) specimens were still unclassified using the two detective methods. In summary, a total of twenty serotypes of enteroviruses including EV71 and CVA16 were identified in these samples. The most prevalent serotypes were EV71 (80, 20.00%), CVA16 (48, 12.00%), CVA10 (73, 18.25%), CVA6 (63, 15.75%), CVA12 (8, 2.00%), CVA2 (5, 1.25%), Echo3 (4, 1%), CVA4 (3, 0.75%), CVB1 (3, 0.75%), CVA9 (2, 0.50%), CVB3 (2, 0.50%) and Echo6 (2, 0.5%). The detailed serotypes of enteroviruses were shown in Fig 1.

A total of 17 cases identified as mixed-infections with at least two serotypes of enteroviruses were observed by GeXP assay, such as the mixed infections of EV71 and CVA16, Echo6 and CVA6, CVA10 and CVA6, CVA4 and Echo6, EV71 and CVB1, EV71,CVA16 and CVA10. The enteroviruse serotypes of mixed-infection varied among these seventeen samples. The female-to-male ratio was 1.43:1. All children were beyond 5 years old.

Among 400 recruited cases, four patients were diagnosed as severe HFMD and 11 patients diagnosed as clustered cases. With regard to 4 severe cases, 2 were EV71 positive, one was CVA10 positive, and one was mixed-infection (EV71, CVA16 and CVA10). The results also showed that 5 out of 11 clustered cases were CVA6 positive, 4 were CVA12 positive and one was Echo24 positive.

**Fig 2:**
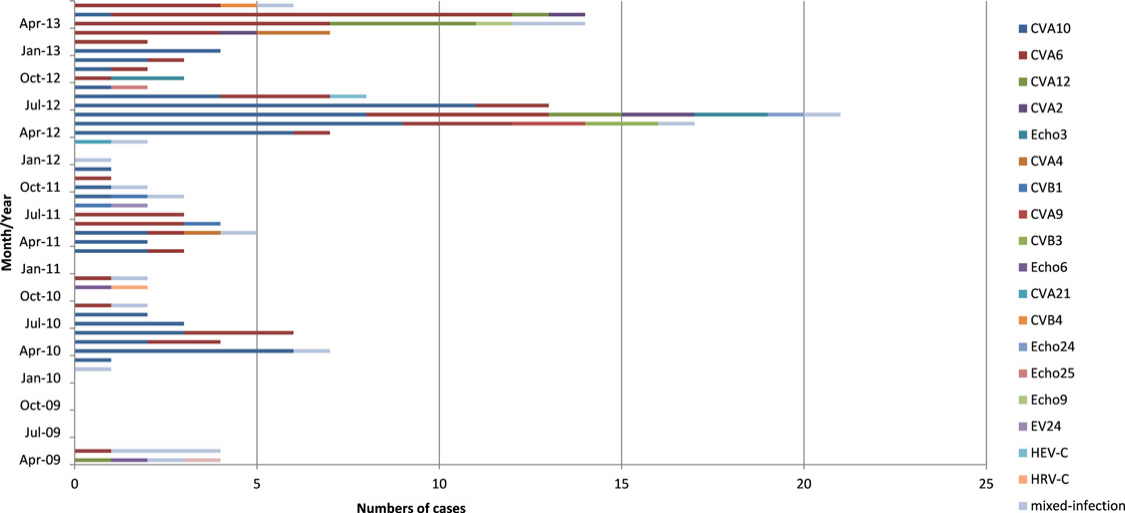
Monthly distribution of non-EV71 and non-CVA16 enterovirus serotypes during 2009 to June 2013 in Jinan, China.

The results in Fig 2 showed that more serotypes of enteroviruses associated with HFMD appeared in spring and summer than in autumn and winter (spring: April-June; autumn: September-November). For examples, 6 serotypes (CVA10, CVA6, CVA12, CVA2, Echo3 and Echo24) of non-EV71 and non-CVA16 enteroviruses co-existed in June 2012, 4 serotypes (CVA10, CVA6, CVA12 and CVA2) of non-EV71 and non-CVA16 enteroviruses in May 2013, 4 (CVA10, CVA6, CVA9 and CVB3) in May 2012 and 3 (CVA10, CVA6 and CVA4) in May 2011. But only one or two serotypes of enteroviruses were prevalent in winter season (Fig 2). Although the serotypes of enteroviruses associated with HFMD varied significantly with years, CVA10 and CVA6 remained the most frequent serotypes of non-EV71 and non-CVA16 every year (with the exception of 2009 in which CVA10 was not found)(Fig 2).

### Distribution of CVA6 and CVA10

**Fig 3:**
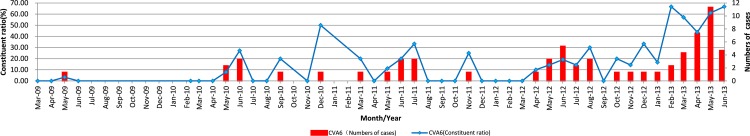
Monthly distribution of CVA6 associated with HFMD during 2009 to June 2013 in Jinan, China.

The results showed that coxsackievirus A6 (CVA6) mainly afflicted children younger than 4 years (56 cases, 88.89%) and the attack rates were highest in 2 year old children (18 cases, 28.57%). The male to female ratio was 2.32:1. CVA6 was increased dramatically from 2009 to June 2013. The proportion of CVA6 infections among the total enterovirus serotypes accounted for 1.32% in 2009, 8.64% in 2010, 11.69% in 2011, 15.45% in 2012 and 51.79% in the first half of 2013. The prevalent seasons for CVA6 were spring and summer followed by a small peak in autumn and early winter (spring: April-June; autumn: September-November) [1] (Fig 3).

**Fig 4:**
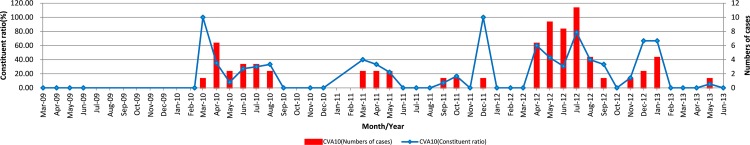
Monthly distribution of CVA10 associated with HFMD during 2009 to June 2013 in Jinan, China.

In this study, CVA10 was most common in children aged one to two (47 cases, 64.38%) and the peak occurred in two years old (31cases, 42.47%). The male to female ratio was 1.52:1. As for CVA10, the circulating trends showed a shift which had higher incidence rate in 2010 (20.99%) and 2012 (38.18%) and lower incidence rate in 2011(11.69%) and the first half of 2013(8.93%). No CVA10- infected case was detected in 2009. The temporal distribution and peak timing of CVA10 were similar to CVA6 which activated in spring and summer, then a slight peak in autumn and early winter (Fig 4).

### Genotyping of 5’-UTR region

**Fig 5:**
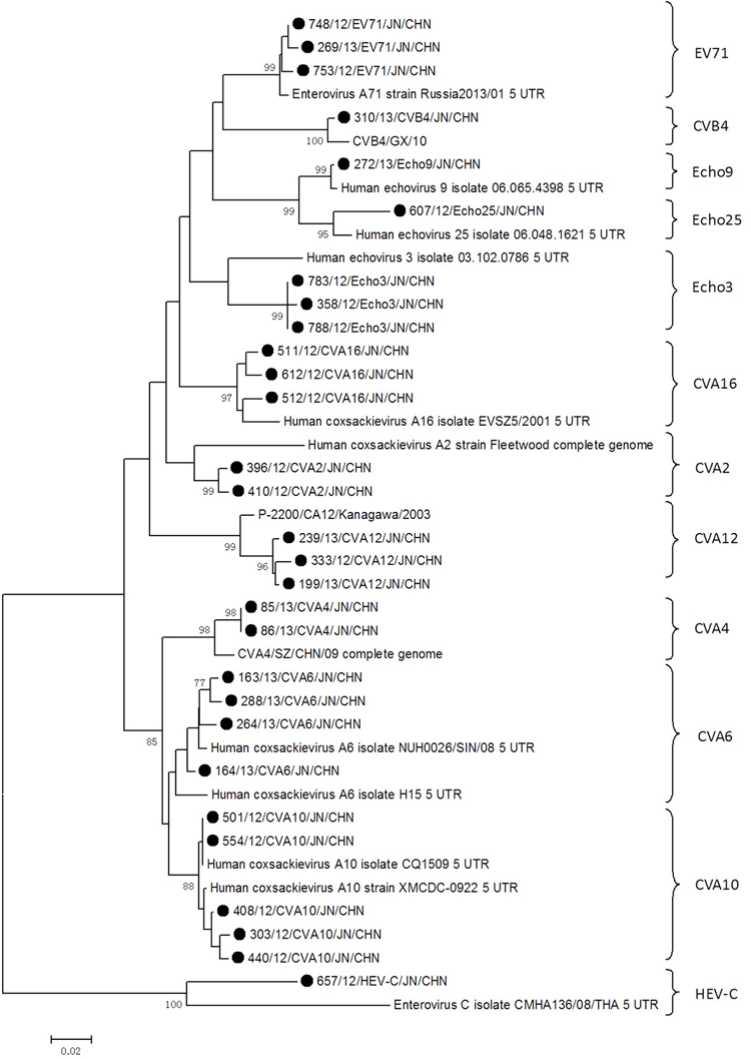
Phylogenetic analysis based on the 5’-UTR region of HFMD cases during 2009 to June 2013 in Jinan, China. A total of 29 representative sequences (representing 12 serotypes) from this study and 12 reference strains worldwide were used to build the tree. Jinan sequences were labeled by black solid dot. Abbreviations: JN, Jinan; CHN, China.

A total of 29 sequences (representing 12 serotypes) on the basis of 5’-UTR region were selected to construct a phylogenetic tree for comparison analysis (Fig 5). The reference strains of corresponding serotypes were downloaded from the GenBank database. The phylogenetic tree was constructed by the neighbor-joining method, and the bootstrap values (1000 replications) were indicated at the branch nodes. Sequence analysis of the 5’-UTR region could effectively discriminate between various enterovirus serotypes such as CVA (2, 4, 6, 10, 16), CVB4, Echo (3, 9, 25), HEV-C and HRV-C detected in our study.

## Discussion

Jinan is the political, cultural and educational center in Shandong province, China. The predominant pathogens causing HFMD have been EV71 and CVA16 from 2009 to 2013 in Jinan city, since HFMD was included into National Notifiable Disease surveillance system in 2008 [8–9]. A certain proportion of non-EV71, non-CVA16 and pan-enterovirus (PE) positive cases associated with HFMD were reported during 2009–2012 in Jinan. In this study, a total of twenty different serotypes of enteroviruses were revealed by GeXP assay combined with nested RT-PCR and sequencing on 5’-UTR region among 400 specimens. Previous study indicated that other enteroviruses were frequently detected in specimens collected from patients who presented with HFMD. For instance, CVA10 were isolated from patients with HFMD in New Zealand in 1957 [19]. The first report of HFMD caused by CVA6 was documented in Finland in 2008, after that, reports of outbreaks caused by CVA6 have increased worldwide such as in Taiwan, France and Guangzhou [20–22]. CVA6, CVA10 and CVA16 were reported the major viral pathogens of HFMD in Singapore [23]. In our study, CVA10 and CVA6 were identified as the main pathogens causing the HFMD other than EV71 and CVA16, which indicated that they were prevalent in Jinan during 2009–2013. In addition, this study also demonstrated the presence of multiple infrequently detected pathogens such as CVA (2, 4, 9, 12, 21), CVB (1, 3, 4), Echo (3, 6, 9, 24, 25), EV24, HEV-C and HRV-C. Such diversities of serotypes of non-EV71 and non-CVA16 enteroviruses associated with HFMD were rarely reported in other previous studies. It is probably due to the methods that did not discriminate all these different viruses and serotypes.

Although all samples collected in this study have been tested to be non-EV71, non-CVA16 and PE positive by the means of RT-PCR or real time PCR, a certain proportion of EV71 (80 cases) and CVA16 (48 cases) were still identified by GeXP assay and confirmed to be true positives by nested RT-PCR and genotyping on 5’-UTR region, indicating that there is a clear lack of a reliable testing method for EV71 and CVA16 and the higher sensitivity and specificity of GeXP assay than that of common RT-PCR test as shown in our previous study [17]. In this study, the proposed GeXP assay was able to identify 9 out of 15 enterovirus serotypes panel including EV71, CVA16, 6, 10, 4 and 9, CVB1 and 3, and ECHO6. The nested RT-PCR and genotyping on 5’-UTR region allowed to classify additional 11 enterovirus serotypes including CVA12, 2 and 21, CVB4, ECHO3, 24, 25 and 9, HEV-C, EV24 and HRV-C besides EV71, CVA16, CVA6 and CVA10, suggesting that 5’-UTR amplification and sequencing provides a powerful supplement to GeXP assay and ensures good genotyping accuracy for clinical samples though only 5 enterovirus serotypes (EV71, CVA16, CVA6, CVA10 and CVA5) were identified from 44 suspected HFMD patients in the original report of genotyping on 5’-UTR region[18]. Our results also showed that 82 samples failed to be classified by both GeXP and 5’-UTR amplification sequencing. The main reasons might be attributed to RNA degradation of partial samples and the gene mutation or recombination of enteroviruses which was frequently observed [18]. Further studies with next-generation sequencing (NGS) are required to clarify the serotypes of these missed samples.

A proportion (17 cases) of mixed-infection specimens with at least two serotypes of enteroviruses were detected through GeXP assay in the present study. GeXP analysis indicated that at least two fragment peaks were observed in mixed-infection specimens. In previous study, mixed-infections were merely reported involving EV71/CVA6 in China, CVA10/CVB1 in Spain [24–25], but other combinations of mixed-infection were rarely detected. In this study, seven serotypes of non-EV71 and non-CVA16 enteroviruses including CVA6, CVA10, CVA4, Echo6, Echo11, CVB1 and CVA5 were identified in mixed-infection specimens. The association of progress of HFMD with mixed-infections of enteroviruses needs to be further addressed after more cases and clinical data are collected in the future.

Previous study demonstrated that CVA6 and CVA10 were becoming important pathogens causing HFMD in Shandong [26], but the epidemiological profiles on them were still lacking in Jinan. CVA6 has attracted more attention probably due to its association often with more severe skin findings and onychomadesis that occurred approximately 1–2 months after infection [6, 27]. In our study, CVA10 and CVA6 were the third and fourth most prevalent pathogens in Jinan, respectively. The epidemiological characteristics of CVA10 and CVA6 were elaborated. Similar to EV71 and CVA16, the attack rates were highest in two years old children and boys had less resistance compared to girls [9]. The seasonal pattern of peaks for CVA10 and CVA6 was in spring/summer and autumn/early winter which was different from Shanghai where the detection frequency of CVA6 and CVA10 changed along with the seasons and reached the peak in winter[28]. CVA6 increased gradually from 2009 to July 2013 and this epidemic trend was also found in Guangzhou from 2010 to 2012 [6]. The incidence of CVA10 showed apparent shift which was high in 2010 and 2012, though low in 2011 and the first half of 2013. It has been indicated that meteorological factors, such as daily mean temperature, relative humidity and duration of sunshine, might play an important role in HFMD epidemiology [29], and it might also apply to infections of CVA10 and CVA6. These epidemiological features of CVA10 and CVA6 could be very helpful in alerting the relevant control agencies for predicting epidemics in the future. This study also indicated that enteroviruses were very active in warm season in terms of the numbers of cases and enteroviruses associated with HFMD.

EV71 infections have always been predominant in severe cases in Jinan since 2008. In this study, one severe case was found to be CVA10 positive. Although no more such severe cases were recruited in this study, Lu et al have reported that CVA10 might be independently correlated with high risk of severe HFMD [26], although with a less effect than that of EV71. No significant differences were identified between CVA10 and EV71 in terms of clinical manifestations. In our study, other enteroviruses such as CVA6 and CVA12 were also found to be associated with clustered cases. Therefore, comprehensive etiological surveillance to detect more serotypes concurrently is necessary and physicians should be aware of these emerging pathogens.

In conclusion, as multiple non-EV71 and non-CVA16 enteroviruses are increasingly contributed to epidemics of HFMD in China, comprehensive surveillance and investigation of pathogen spectrum in the prevalent seasons are significantly important and will improve our understanding, prevention and control of HFMD. Based on the results obtained from this study, GeXP assay combined with nested RT-PCR on 5’-UTR region has the potential to be widely used as a high throughput, robust, and reproducible method in the surveillance of diversity of non-EV71 and non-CVA16 enteroviruses associated with HFMD. Although 20% samples could not be detected virus presence in this study, NGS would perhaps provide a powerful supplement to GeXP and nested RT-PCR assay for unclassified cases in the future.
